# Supplementary material for: Use of Low-Dose Tamoxifen to Increase Mammographic Screening Sensitivity in Premenopausal Women
Source: Cancers (Basel). 2021 Jan 15;13(2):302. doi: 10.3390/cancers13020302 (PMC7830894; doi:10.3390/cancers13020302)
Supplement: Supplementary file 1 [file cancers-13-00302-s001.pdf]

# Supplementary Material: Use of Low-Dose Tamoxifen to Increase Mammographic Screening Sensitivity in Premenopausal Women

**Mikael Eriksson, Kamila Czene, Emily F. Conant and Per Hall**

**Text S1.** KARISMA trial synopsis.

It is known that tamoxifen prevents breast cancer in high-risk women and reduces mortality in the adjuvant setting tamoxifen but comes with side-effects. The optimal dose to increase uptake in the population is unknown. A reduction of mammographic density is an early marker of response to tamoxifen.

The KARISMA trial (Trial Registration: EudraCT 2016-000882-22, clinicaltrials.gov) objective was to determine if low-dose tamoxifen is non-inferior to standard dose to lower mammographic density and if low-dose tamoxifen is associated with fewer symptoms. The trial design was a six-months double-blind placebo-controlled randomized dose-determination trial in 2016–2019. Mammographic density changes were measured using the fully automated STRATUS density tool. Menopausal-like symptoms were assessed using an on-line self-report questionnaire.

The KARISMA study participants were healthy pre- and postmenopausal women aged 40 to 74 years participating in the Swedish population-based mammography screening program who volunteered to participate. $N = 2314$ screening women were investigated for eligibility. The exclusion criteria were women with a low mammographic density (BI-RADS A), high blood pressure, pregnancy, use of hormonal therapy, previous cardiovascular disorder, uncontrolled diabetes, and any previous cancer. The women received six months oral daily tamoxifen dose of 1, 2.5, 5, 10, and 20 mg of tamoxifen or placebo (Diagram 1).

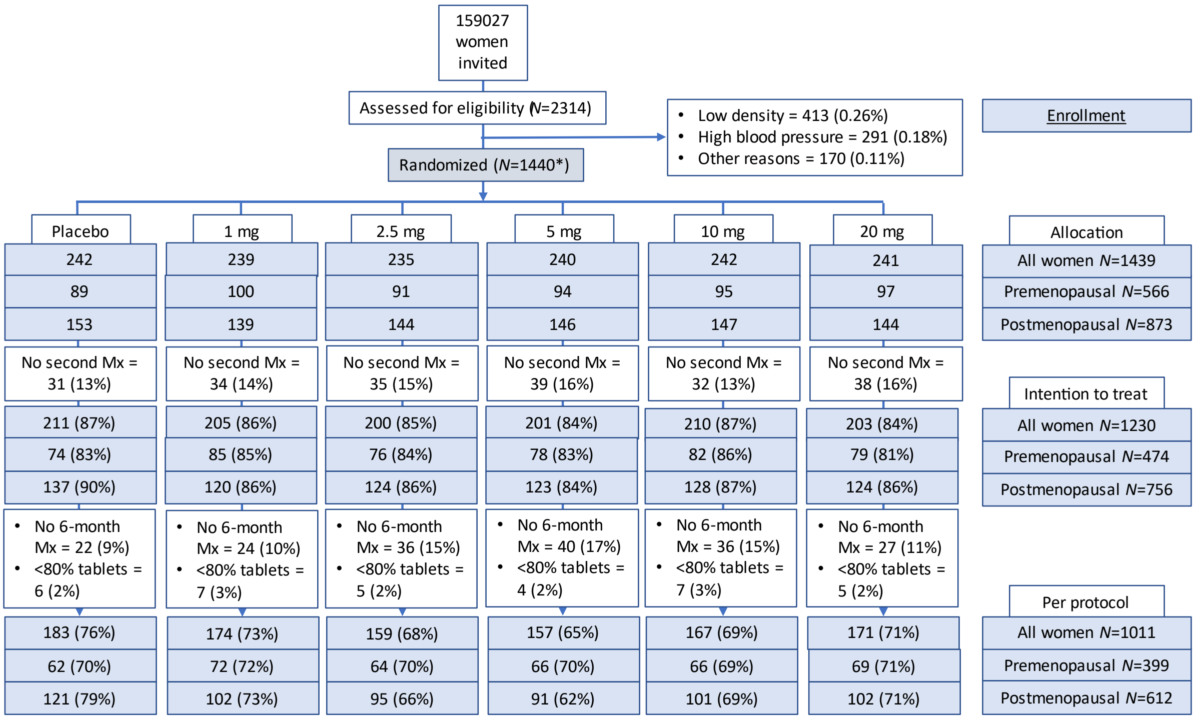


* One premenopausal woman was later found to have no measurable density and was excluded, leaving 1439 in the study

**Diagram 1:** Consort diagram of KARISMA. Mx = mammogram.

The trial outcomes were non-inferior reductions in mammographic density and fewer severe symptoms in lower doses compared with the standard dose 20 mg. Post-hoc analyses were performed in of the outcomes by menopause status. Mammographic density was assessed as radiographic dense fibro-glandular tissue. The assessed symptoms included vasomotor, gynecological, sexual, and joint pain symptoms. Intention to treat and per protocol populations were analyzed.

In all, 1440 pre- and postmenopausal participants were recruited to the study, 240 women in each arm. The premenopausal women showed non-inferior reduction in mammographic density following 2.5, 5 and 10 mg tamoxifen use compared with the median 9.6% decrease observed in the 20 mg tamoxifen group. No reduction was seen in the 1 mg arm. No reduction in breast density was seen in the postmenopausal participants. Severe vasomotor symptoms (hot flashes, cold and night seats) were reduced with approximately 50% in the 2.5 mg group compared with the 20 mg group.

In conclusion, premenopausal women showed fewer side effects with non-inferior magnitude of breast density decrease at lower dose of tamoxifen (2.5 mg) compared with standard dose (20 mg). A low dose of tamoxifen could be used for prevention and increasing sensitivity of a mammogram in premenopausal women. The Study Protocol is available on-line.

**Table S1.** Association between density response due to tamoxifen and breast cancer risk factors at baseline in KARISMA.

| Risk Factor | Density Response | | | | | |
|---|---|---|---|---|---|---|
| | Linear Model | | | Quadratic Model | | |
| | **Beta** | ***p*-Value** | **Holm *p*-Value** | **Beta** | ***p*-Value** | **Holm *p*-Value** |
| Baseline percent mammographic density | 0.000 | 0.85 | 0.94 | 0.000 | 0.92 | 0.99 |
| Baseline mammographic dense area ($cm^2$) | −0.001 | 0.61 | 0.94 | 0.000 | 0.79 | 0.99 |
| Age | −0.001 | 0.91 | 0.94 | −0.001 | 0.58 | 0.99 |
| BMI | 0.001 | 0.94 | 0.94 | 0.003 | 0.19 | 0.99 |
| Age at menarche | 0.014 | 0.71 | 0.94 | −0.004 | 0.84 | 0.99 |
| Parity | 0.131 | 0.56 | 0.94 | 0.000 | 0.99 | 0.99 |
| Age at first birth | 0.022 | 0.03 | 0.25 | 0.000 | 0.78 | 0.99 |
| Regular smoking during last year | −0.087 | 0.27 | 0.84 | −0.149 | 0.27 | 0.99 |
| Regular alcohol drinking during last year | −0.001 | 0.34 | 0.84 | 0.000 | 0.67 | 0.99 |
| Breast cancer in family | −0.162 | 0.13 | 0.67 | 0.000 | 0.99 | 0.99 |

KARISMA = Karolinska intervention trial of low-dose tamoxifen. In the 2.5 mg tamoxifen arm of the premenopausal women in the KARISMA trial the table presents the association between density response and breast cancer risk factors at baseline. Results are presented as beta coefficients from linear and quadratic univariable models. Density response is defined as relative density change at follow-up compared to baseline. The Holm *p*-values presents adjusted *p*-values using the Holm–Bonferroni multiple comparison statistic based on the family of risk factors. The beta coefficients in the quadratic model is from the multiplication term.

**Table S2.** Characteristics of mammographic density response in the KARMA exposed group including 28,282 premenopausal women.

| Mammographic Density Response Characteristics | All Women | Non-Breast Cancers | Screen Detected Cancers | Interval Cancers |
|---|---|---|---|---|
| Number of women | 28,282 | 27,765 | 287 | 230 |
| Mammographic density at baseline | | | | |
| Percent mammographic density, mean (SD) | 26.6 (22.9) | 26.5 (22.9) | 25.3 (21.5) | 33.0 (24.0) |
| Mammographic dense area $cm^2$, mean (SD) | 32.4 (28.5) | 32.3 (28.5) | 33.5 (27.8) | 41.8 (35.4) |
| Mammographic density after exposure | | | | |
| Relative percent density change, mean % | −14.8 | −14.8 | −18.7 | −15.8 |
| Relative percent density change, median % | −21.9 | −21.9 | −22.7 | −21.9 |
| Relative dense area $cm^2$ change, mean % | −15.4 | −15.4 | −19.4 | −16.4 |
| Relative dense area $cm^2$ change, median % | −20.8 | −20.8 | −23.1 | −20.8 |
| Distribution of BI-RADS density categories[1] | | | | |
| A, % | 7 | 7 | 6 | 3 |
| B, % | 33 | 33 | 36 | 21 |
| C, % | 47 | 47 | 46 | 58 |
| D, % | 13 | 13 | 12 | 18 |
| Density response categories | | | | |
| <10% decrease, % | 28 | 28 | 25 | 26 |

| Mammographic Density Response Characteristics | All Women | Non-Breast Cancers | Screen Detected Cancers | Interval Cancers |
|---|---|---|---|---|
| 10-<20% decrease, % | 17 | 17 | 16 | 21 |
| 20-<40% decrease, % | 39 | 39 | 35 | 39 |
| 40-<60% decrease, % | 9 | 9 | 14 | 10 |
| 60-<80% decrease, % | 5 | 5 | 7 | 3 |
| ≥80% decrease, % | 2 | 2 | 2 | 2 |
| | Density responder cut-offs | | | |
| ≥10% decrease, % | 72 | 72 | 75 | 74 |
| ≥20% decrease, % | 55 | 55 | 58 | 53 |
| ≥30% decrease, % | 27 | 26 | 31 | 26 |
| ≥50% decrease, % | 11 | 11 | 16 | 7 |

SD = standard deviation. KARMA = Karolinska mammography project for risk prediction of breast cancer. [1]Computer-generated BI-RADS breast composition categorization. In the KARMA exposed group, the table presents characteristics of mammographic breast density response in the exposed group stratified by women without breast cancer and with breast cancer by screening and interval cancer status.

**Table S3.** Sensitivity analysis of number of interval cancers per 100,000 age standardized screened premenopausal women in the unexposed group and in the exposed group by percentage of relative mammographic density decrease.

| Number of Interval Cancers | Unexposed Group (*N*) | Density Responder Cut-Off (%) | | | |
|---|---|---|---|---|---|
| | | ≥10 | ≥20 | ≥30 | ≥50 |
| BI-RADS density category | - | Exposed group (*N*) | | | |
| A+B | 155 | 107 | 104 | 106 | 91 |
| C | 382 | 358 | 343 | 298 | 268 |
| D | 276 | 202 | 194 | 177 | 150 |
| A to D combined | 813 | 667 | 641 | 581 | 509 |
| - | | Difference compared to unexposed group, *N* (%) | | | |
| A+B | ref. | −48 (−31) | −51 (−33) | −49 (−32) | −64 (−41) |
| C | ref. | −24 (−6) | −39 (−10) | −84 (−22) | −114 (−30) |
| D | ref. | −74 (−27) | −82 (−30) | −99 (−36) | −126 (−46) |
| A to D combined | ref. | −146 (−18) | −172 (−21) | −232 (−29) | −304 (−37) |

KARISMA = Karolinska intervention study of low-dose tamoxifen. In the KARMA exposed group, the table presents sensitivity analysis of the number of interval cancers per 100,000 age standardized screened premenopausal women together with change in number of interval cancers in the exposed group by percentage mammographic density decrease. The density responses from the 2.5, 5, 10, 20 mg arms in the KARISMA trial were used. Density response is presented using density responder cut-offs and is stratified by computer-generated BI-RADS categories A+B, C, D. The unexposed group is included as the reference. *N* = 265 premenopausal women from the 2.5, 5, 10, 20 mg arms in the KARISMA trial were used as the reference for density response to tamoxifen in KARMA.

**Table S4.** Sensitivity analysis in full KARMA cohort of menopause status modification of the screening sensitivity and the tumor size dependence of mammographic density.

| Characteristic | *p*-Value |
|---|---|
| Screening sensitivity | 0.37 |
| Tumor size (± 20 mm) | 0.79 |

The table presents how menopause status affects how screening sensitivity and tumor size is associated with mammographic density. The screening sensitivity logistic model was fit using interval cancer status as dependent variable, mammographic density as independent variable, adjusted for year of mammogram. The tumor size logistic model was fit after exchanging screening sensitivity with tumor size.

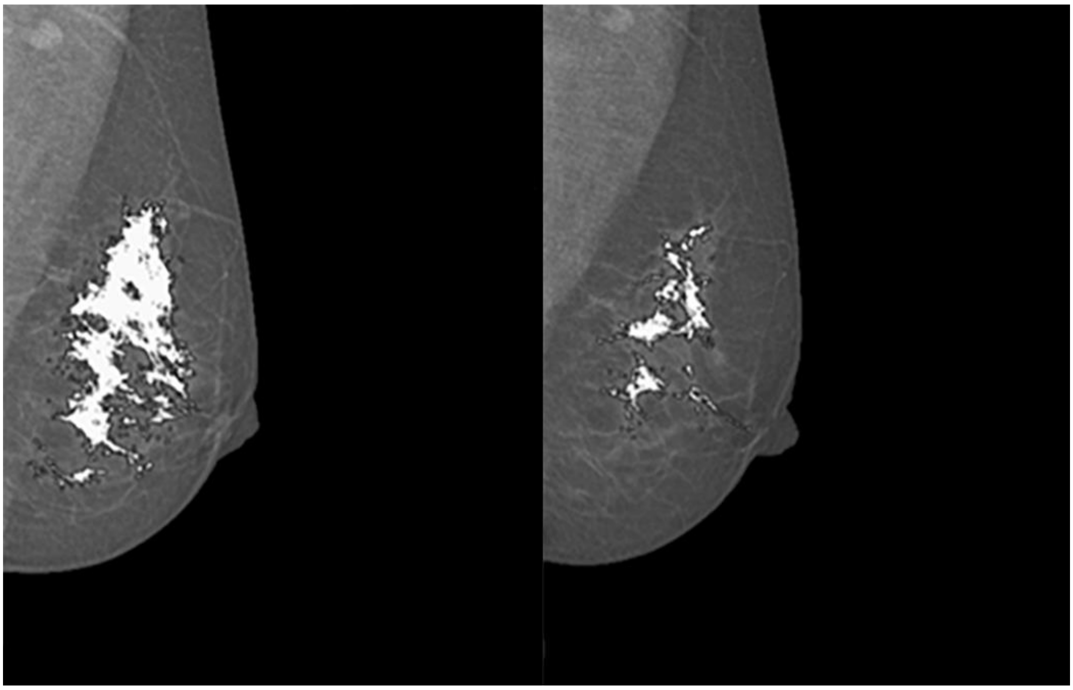

**Figure S1.** Mammographic density response to 6 months use of tamoxifen in the KARISMA trial. Two mammograms of a woman participating in the KARISMA trial. One mammogram at baseline and one mammogram after 6 months of tamoxifen exposure. The percent mammographic density was 42% at the baseline mammogram and was reduced to 14% percent density at the 6 months mammogram.

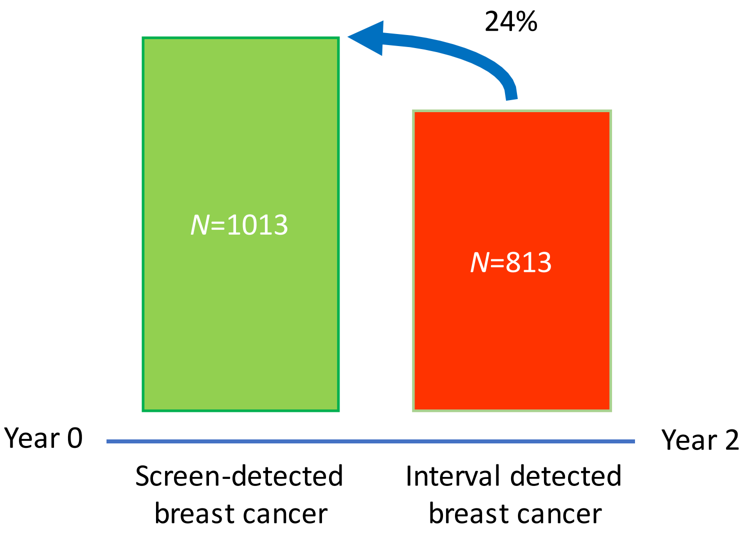


**Figure S2.** Estimated reduction of interval cancers per 100,000 age standardized screened premenopausal women after six months exposure to low-dose tamoxifen and ≥20% relative mammographic density reduction. In the KARMA unexposed group, the figure presents screen-detected breast cancers ($N = 1013$) and interval breast cancers ($N = 813$) per 100,000 age standardized premenopausal women. After six months exposure to low-dose tamoxifen 24% of the interval cancers were found in the group of women that had a relative reduction of mammographic density by ≥20%.

**Publisher's Note:** MDPI stays neutral with regard to jurisdictional claims in published maps and institutional affiliations.

© 2021 by the authors. Licensee MDPI, Basel, Switzerland. This article is an open access article distributed under the terms and conditions of the Creative Commons Attribution (CC BY) license (http://creativecommons.org/licenses/by/4.0/).
